# CD20 is dispensable for B-cell receptor signaling but is required for proper actin polymerization, adhesion and migration of malignant B cells

**DOI:** 10.1371/journal.pone.0229170

**Published:** 2020-03-25

**Authors:** Veronika Kozlova, Aneta Ledererova, Adriana Ladungova, Helena Peschelova, Pavlina Janovska, Aleksander Slusarczyk, Joanna Domagala, Pavel Kopcil, Viera Vakulova, Jan Oppelt, Vitezslav Bryja, Michael Doubek, Jiri Mayer, Sarka Pospisilova, Michal Smida

**Affiliations:** 1 Central European Institute of Technology (CEITEC), Masaryk University, Brno, Czech Republic; 2 Department of Internal Medicine - Hematology and Oncology, Medical Faculty of Masaryk University and University Hospital Brno, Brno, Czech Republic; 3 Department of Experimental Biology, Faculty of Science, Masaryk University, Brno, Czech Republic; 4 Department of Immunology, Medical University of Warsaw, Warsaw, Poland; University of Illinois at Chicago, UNITED STATES

## Abstract

Surface protein CD20 serves as the critical target of immunotherapy in various B-cell malignancies for decades, however its biological function and regulation remain largely elusive. Better understanding of CD20 function may help to design improved rational therapies to prevent development of resistance. Using CRISPR/Cas9 technique, we have abrogated CD20 expression in five different malignant B-cell lines. We show that CD20 deletion has no effect upon B-cell receptor signaling or calcium flux. Also B-cell survival and proliferation is unaffected in the absence of CD20. On the contrary, we found a strong defect in actin cytoskeleton polymerization and, consequently, defective cell adhesion and migration in response to homeostatic chemokines SDF1α, CCL19 and CCL21. Mechanistically, we could identify a reduction in chemokine-triggered PYK2 activation, a calcium-activated signaling protein involved in activation of MAP kinases and cytoskeleton regulation. These cellular defects in consequence result in a severely disturbed homing of B cells *in vivo*.

## Introduction

Protein CD20 on the surface of B cells is used in the clinic as a key target of therapy with monoclonal antibodies to treat B-cell malignancies [1]. However, its expression often declines in response to anti-CD20 monoclonal antibodies (mAbs), thereby mitigating any additional impact of their repeated administration [2]. Furthermore, CD20 expression is highly variable and rather low on the cells of chronic lymphocytic leukemia (CLL) when compared to other B-cell malignancies or healthy B cells [3]. Notably, some experimental approaches to pharmacologically modulate CD20 expression levels were proposed, nevertheless, none of the strategies has ever been transferred into the clinical use [4, 5]. So far, it is impossible to predict CD20 expression changes, as the precise molecular mechanisms of CD20 regulation and its biological function remain largely elusive.

CD20 is a tetraspanin membrane protein solely expressed on the surface of B cells. Primarily, it was proposed to play a role as a calcium channel [6, 7], as CD20 ectopic overexpression was able to generate calcium influx into the cells [7, 8]. To date, its involvement in the B-cell receptor (BCR) signaling remains contradictory. On one hand, some evidence showed CD20 to directly associate with BCR, to be involved in BCR signaling and to regulate cell cycle progression of B cells [6, 9–11]. Partial downregulation of CD20 with a single siRNA exhibited some minor effect upon BCR signaling [7, 11]. On the contrary, results from CD20 knockout mice demonstrated that CD20 is fully dispensable for both proper BCR signaling and normal B cell proliferation [12, 13]. Conclusive genetic experiments in human cells are so far missing. In order to provide clear genetic evidence for the consequences of CD20 loss in malignant B cells, we make use of the CRISPR/Cas9 system to disrupt CD20 gene in total of five malignant B cell lines. Functional analyses are performed to assess the propensity of BCR signaling, chemokine signaling and cell behavior of CD20-knockout cells.

## Materials and methods

### Cell culture

Burkitt lymphoma cell lines Ramos and Raji were obtained from ATCC, diffuse large B-cell lymphoma cell line Oci-Ly1 and chronic lymphocytic leukemia cell line MEC1 were obtained from DSMZ. Chronic lymphocytic cell line HG3 was kindly provided by the lab of Dr. Rosenquist (Uppsala, Sweden). Ramos, Raji, Oci-Ly1 and HG3 cells were cultured in RPMI 1640 (HyClone, GE Healthcare), while MEC1 cell line was cultured in IMDM (HyClone, GE Healthcare) medium. Media were supplemented with 10% heat-inactivated fetal bovine serum (FBS; Biosera) and penicillin/streptomycin (Sigma-Aldrich). Cell cultures were maintained at 37 °C in 5% CO_2_ atmosphere.

### Generation of CD20 knockout cells

GuideRNA (sgRNA) targeting the first coding exon within *MS4A1* gene was designed (target sequence: TGCTAGCTGCGGAGTTCAGT) using the online tool crispr.mit.edu and cloned into LentiGuide-puro vector (Addgene, # 52963) according to the established protocol [14, 15]. Correct cloning was verified by Sanger sequencing. Lentiviral particles were produced by transfecting HEK293T cells (ATCC) with cloned CD20 sgRNA construct together with VSV.G and dR8.91 lentiviral vectors using polyethylenimine PEI MAX (Polysciences). Lentiviruses were collected 48–72 hours post transfection and used to transduce MEC1, Ramos and HG3 cells previously infected with lentiCas9-blast vector (Addgene, # 52962). Following antibiotic selection, cells were cultured for additional week to allow CD20 depletion and CD20-negative cells were sorted out on a FACS sorter FACSAria Fusion (BD Biosciences). Cells expressing Cas9 alone were used as control cell lines throughout the study.

To generate CD20 knockouts in Oci-Ly1 and Raji cells, sgRNA oligonucleotides containing sgCD20_1 (5’-CACCGGGATCATCAGAAGACCCCCCGTTT-3‘) or sgGFP (5’-GGGGGAGGAGCTGTTCACCG-3’) (used as a negative control) were cloned into the lentiCRISPR-V2 vector (Addgene, # 52961). For lentiviral production, HEK-293T cells were co-transfected with lenti-CRISPR-V2 vectors together with psPAX.2 and pMD2.G plasmids using PEG. After 72h lentivirus-containing medium was filtrated through 0,45 μm filter and added to Raji and OCI-Ly1 cells. After transduction, puromycin (1 μg/ml) was added to culture medium for the following week as a selection antibiotic to obtain stable cell lines. Finally, CD20-negative cells were sorted with FACSAria III using Diva software (Beckton Dickinson, Franklin Lakes, New Jersey, USA).

### Analysis of CRISPR off-target sites

Potential off-target sites for CD20 CRISPR sgRNA were predicted using the online tool crispr.mit.edu. Top three predicted exonic off-target sites were amplified from the CD20 knockout and control cells by PCR using specific primers (AKAP9—F: CATGCGAAAGTGACACAGACA, R: GATTGACGGCTTCCAAACCT; BOC—F. CAGAAGAGCATCCAGAGCCAT, R: GAAGCTTTCAGGTGCCCTGTT; SETD6 –F: AGATGAGAGAGGAAATGTGGGAT, R: TGTAAGGCTTGCTGTTCCCT). PCR products were cleaned up by ExoSAP (Affymetrix) and labeled with BigDye Terminator v1.1 sequencing Kit (ThermoFisher Scientific). Samples were purified by gel filtration through Sephadex resin (Sigma) and sequenced on ABI 3500 Genetic Analyzer (Applied Biosystems). No off-target editing was observed in CD20 knockout cells compared to wild-type counterparts.

### Quantitative real-time PCR

mRNA was extracted from the cells with RNeasy mini isolation kit (Qiagen) according to the manufacturer´s instructions. cDNA was prepared using RevertAid reverse transcriptase (ThermoFisher Scientific) and random hexamer primers (ThermoFisher Scientific). Quantitative real-time PCR was performed in triplicates with the XCeed SYBR Green qPCR 2x mix (IAB) and qPCR primers specific for CD20 (F: CACCCATCTGTGTGACTGTGTG, R: AGTTTTTCTCCGTTGCTGCC) and HPRT (F: GCTATAAATTCTTTGCTGACCTGCTG, R: AATTACTTTTATGTCCCCTGTTGACTGG), which was used as the loading control. Real-time PCR was performed on Quantstudio 12K Flex system (Applied Biosystems).

### Flow cytometry and calcium flux measurement

Cells were washed once with PBS and labeled with human CD20-APC, CXCR4-PE, CCR7-FITC or IgG isotype control antibodies (all from ThermoFisher Scientific) for 20min at 4°C. After one wash with PBS, cells were acquired on FACSVerse (BD Biosciences) and data were analyzed using FlowJo v10.0.7 (TreeStar).

To determine the calcium flux, Ramos CD20 knockout and control cells were loaded with the calcium-sensitive dye Fluo-4 AM (1 μg/ml) mixed 1:1 with Pluronic F-127 (both from ThermoFisher Scientific) and incubated for 30min at 37°C in RPMI 1640 medium without phenol red (Biosera) supplemented with 10% heat-inactivated FBS. The tube was then filled up with RPMI 1640 without phenol red + 10% FBS and incubated for additional 30min at 37°C. Cells were washed and kept on ice until use.

Before the measurement, cells were prewarmed at 37°C for 5min. First, the baseline was recorded for 1min, followed by addition of 10 μg/ml Goat F(ab’)_2_ anti-human IgM-UNLB antibody (SouthernBiotech). Ionomycin (1 μg/ml, ThermoFisher Scientific) was added at 5min as a positive control.

To dissect the intracellular calcium release and the influx across the plasma membrane, we pretreated the cells with 0.5 mM EGTA for 5min at 37°C, measured the baseline for 1min, added 10 μg/ml Goat F(ab’)_2_ anti-human IgM-UNLB antibody (SouthernBiotech) to trigger intracellular calcium release and finally added 1 mM calcium chloride at 4min to assess the influx from extracellular space. Measurements were performed on FACSVerse and data analyzed by FlowJo v10.0.7 (TreeStar) software.

### Dose-response curves

Cells were incubated with a concentration range of CD20-mAbs Rituximab or Obinutuzumab (Roche) in the presence of 20% human serum (purchased from University Hospital Brno) in 96-well plates in triplicates for three days. To assess cell viability, CellTiter-Glo Luminescent Cell Viability Assay (Promega) was performed as recommended by the manufacturer and samples were measured with Tecan Spark 10M system. Acquired values were normalized against PBS-treated wells and plotted as relative viability.

### Cell stimulation and Western blotting

Cells were washed 1x with PBS and incubated in serum-free medium for one hour at 37°C. Next, 2x10^6^ cells per sample were stimulated in serum-free medium by adding 10 μg/ml Goat F(ab’)_2_ anti-human IgM-UNLB antibody (SouthernBiotech) or 10 μg/ml anti-CD20 mAb Rituximab, Ofatumumab (Genmab) or Obinutuzumab together with 3.3 mM H_2_O_2_ (Dr. Kulich Pharma) and incubated for indicated times at 37°C. Stimulation was stopped by adding 1 ml ice-cold Tris-buffered saline (TBS).

Cells were lysed for 20 min on ice in RIPA lysis buffer (50 mM Tris, 1% NP-40, 150 mM sodium chloride (Amresco), 0.1% SDS, 10 mM sodium pyrophosphate (Sigma-Aldrich), 1 mM PMSF, 0.5% sodium deoxycholate (Carl Roth), 1 mM sodium vanadate (Alfa Aesar), 50 mM sodium fluoride (Penta)). Cell lysates were centrifuged at 13,000 rpm, 4°C and supernatants boiled with 4x Laemmli sample buffer (Bio-Rad) at 95°C for 10min. Protein lysates were separated by 10% polyacrylamide gel electrophoresis, transferred onto nitrocellulose membrane (Bio-Rad) and blocked for one hour at room temperature in 5% BSA (Serva) in TBS-Tween20. Broad range (11–245 kDa) color prestained protein standard (NEB) was run alongside the lysates to assess the molecular weight of detected proteins. Membranes were incubated with antibodies against p-PLCγ2 (Tyr1217, #3871), p-CD19 (Tyr531, #3571), p-CD79A (Tyr182, #5173), p-BTK (Tyr223, #5082), p-AKT (Ser473, #4060), p-SYK (Tyr525/526, #2710), p-Src family kinases (Tyr416, #6943), p-LYN (Tyr507, #2731), p-PYK2 (Tyr402, #3291), p-p38 (Thr180/Tyr182, #4511), p-ERK1/2 (Thr202/Tyr204, #4377), p-JNK (Thr183/Tyr185, #4668) (all from Cell Signaling Technology), CD20 (#04–455, Merck Millipore), Actin (#A5441) and Tubulin (#T5168) (both from Sigma-Aldrich). Membranes were washed with TBS-Tween20 and incubated with secondary goat anti-rabbit (#170–6515) or goat anti-mouse (#170–6516) HRP-conjugated antibodies (Bio-Rad). Detection was done using the Clarity Western ECL Substrate (Bio-Rad) and UVITEC Alliance 4.7 detection system.

### Cell cycle analysis and growth curve

To analyze the cell cycle progression, cells were washed once with PBS and fixed in 70% ethanol for 30min at -20°C. After additional wash with PBS, cells were stained in Vindel´s solution (10 mM Tris pH 8.0, 1 mM NaCl, 0.1% Triton X-100, 40 μg/ml RNase A, 50 μg/ml Propidium Iodide) for 30min at 37°C. Samples were measured on FACSVerse and cell cycle profiles analyzed by FlowJo v10.0.7 (TreeStar) software.

To assess potential growth defects, cells were seeded in triplicates at 400,000 cells per well in 6-well plates. Cells were counted every 3–4 days and inoculated again at 400,000 cells/well. Cumulative cell density was plotted on a logarithmic y axis to generate the growth curve.

### RNA sequencing

RNA from triplicate samples was isolated using RNeasy Mini Kit (Qiagen) according to manufacturer’s recommendations. Poly(A) mRNA selection was done from 100 ng total RNA using NEBNext Poly(A) mRNA Magnetic Isolation Module and sequencing library was prepared with NEBNext Ultra II Directional RNA Library Prep Kit for Illumina using NEBNext Multiplex Oligos for Illumina (New England Biolabs) and Agencourt Ampure XP Beads (Beckman Coulter). In brief, poly(A) mRNA enriched samples were fragmented and transcribed into cDNA. Following universal adapter ligation, samples were barcoded using NEB dual indexing primers, library quality was assessed on Fragment Analyser using High Sensitivity NGS Fragment Analysis Kit (Advanced Analyticals) and equimolar amounts were pooled after picogreen quantitation. Sequencing was performed with the NextSeq 500/550 High Output v2 kit on Illumina NextSeq 500 sequencer using 75 cycles, in collaboration with CEITEC Genomics Core Facility.

Acquired sequencing data were quality checked using FastQC [16]. The sequences were trimmed for adaptors and low-quality ends using Trimmomatic tool [17] and the preprocessed reads were aligned onto a human reference genome using STAR [18]. Differential gene expression analysis was performed using DESeq2 package [19]. P-values were adjusted for multiple testing using Benjamini-Hochberg method and genes with an adjusted p-value ≤ 0.05 were considered statistically significantly differentially expressed. Gene ontology enrichment analysis was performed using online tool GOrilla [20].

Original sequencing data are deposited at the NCBI Sequence Read Archive (SRA) under the accession number PRJNA550104.

### Actin polymerization

10^5^ cells were washed once with PBS and stimulated with 100 ng SDF1α or CCL19 chemokines (PeproTech) for 15 seconds at room temperature. Stimulation was quickly stopped by adding mixture of 4% paraformaldehyde and 1% Triton X-100 in PBS. Polymerized actin was visualized by staining with TRITC-phalloidin (ThermoFisher Scientific) for 15min at room temperature in dark. Samples were then measured on FACSVerse.

### Transwell migration and time-lapse microscopy

Migration was done in 24-well plates with transwell inserts having 8-μm pore size (Corning Incorporated). 600 μl medium containing 0.5% BSA and the chemokine (200 ng/ml SDF1α, 200 ng/ml CCL19, 200 ng/ml CCL21 or none; all PeproTech) was prepared in the lower compartment. Cell suspension (5x10^5^ cells in 100 μl) was pipetted into the insert and cells were allowed to migrate for 5 hours. Cells in the bottom part were retrieved, counted with the C6 flow cytometer (Accuri) and calculated as the percentage of the migrated cells versus total number of cells plated into the insert (original cell suspension was counted with C6 cytometer in parallel).

Time-lapse imaging was performed on 24-well plates, which were precoated with 10 μg/ml Fibronectin (Sigma-Aldrich) for 1h at room temperature. 30,000 cells were plated per well and allowed to settle for 1 hour at 37°C before measurement. Imaging was done on Olympus IX83 microscope, with 10x objective, 180 frames every 20 seconds, using Olympus CellSense dimension software. Data were analyzed using ImageJ program and CellTracker ver. 1.1 software. At least 40 cell tracks were analyzed for each sample. Graphs were plotted in GraphPad Prism 7.

### Cell adhesion

96-well plates were coated with 10 μg/ml Fibronectin (Sigma-Aldrich) or 1 μg/ml VCAM (PeproTech) diluted in 2% BSA in PBS without Ca^2+^ and Mg^2+^ overnight at 4°C. Plates were washed 3x with PBS, blocked for 2h in 2% BSA/PBS at 37°C and washed once with Hanks´ Balanced Salt solution (HBSS) (Sigma-Aldrich). 1x10^5^ cells in HBSS were stimulated in triplicates with 200 ng/ml SDF1α, 200 ng/ml CCL19, 2 mM MnCl_2_ or PBS for 15min and then plated onto precoated wells. Cells were allowed to adhere for 30min at 37°C. At the end, non-adhered cells were carefully aspirated and wells carefully washed with HBSS. CellTiter-Glo diluted 1:6 in PBS was added into the wells to measure the amount of adhered cells. Samples were measured using Tecan Spark 10M system. Obtained values were normalized against the CellTiter-Glo measurement of the original input cell suspension and depicted as the percentage of adhered cells relative to the total cell number.

### Homotypic cell adhesion

Cells were resuspended in complete medium, plated on 24-well flat-bottom plates and 10 μg/ml anti-HLA-DR (L243, Exbio) or anti-CD44 (MEM-85, Exbio) antibodies was added. Untreated cells received PBS instead of antibodies. Cells were cultured for 6h (MEC1 cells) or 24h (Ramos) at 37°C and the photographs were taken by Olympus inverted microscope using a 10x objective (Olympus).

### *In vivo* cell homing

5x10^6^ control or CD20-knockout cells was loaded with Calcein AM (ThermoFisher Scientific) according to the manufacturer´s recommendation. Cells were injected in 200 μl sterile PBS into the non-irradiated nonobese diabetic/severe combined immunodeficient (NOD/SCID) IL2Rγ-null (NSG) mice (obtained from the Jackson Laboratory). MEC1, Raji and Ramos cells were administered intraperitoneally and mice were sacrificed by cervical dislocation 24 hours post injection. HG3 cells were injected intravenously into the tail vein and mice sacrificed 6 hours later. Blood, spleen, liver and bone marrow were excised, mechanically minced and single cell suspensions were filtered through cell strainers (VWR). The percentage of calcein-positive cells was determined by FACS analysis (BD Biosciences). The animal experiments were performed with the approval of the Ethics committee for research of the Masaryk University (approval nr. EKV-2017-008) according to the Law on the Protection of Animals against Cruelty (Act No. 246/1992 Coll.).

### Statistical analysis

All experiments were repeated at least 3 times unless specified otherwise. To assess statistical significance, unpaired T-test was calculated in GraphPad Prism 7 for single comparison. Two-way ANOVA with Sidak´s correction for multiple comparisons testing was used to compare samples in experiments with multiple conditions. Significance was determined as follows: not significant p > 0.05, * p < 0.05, ** p < 0.01, *** p < 0.001, **** p < 0.0001.

## Results and discussion

**Fig 1:**
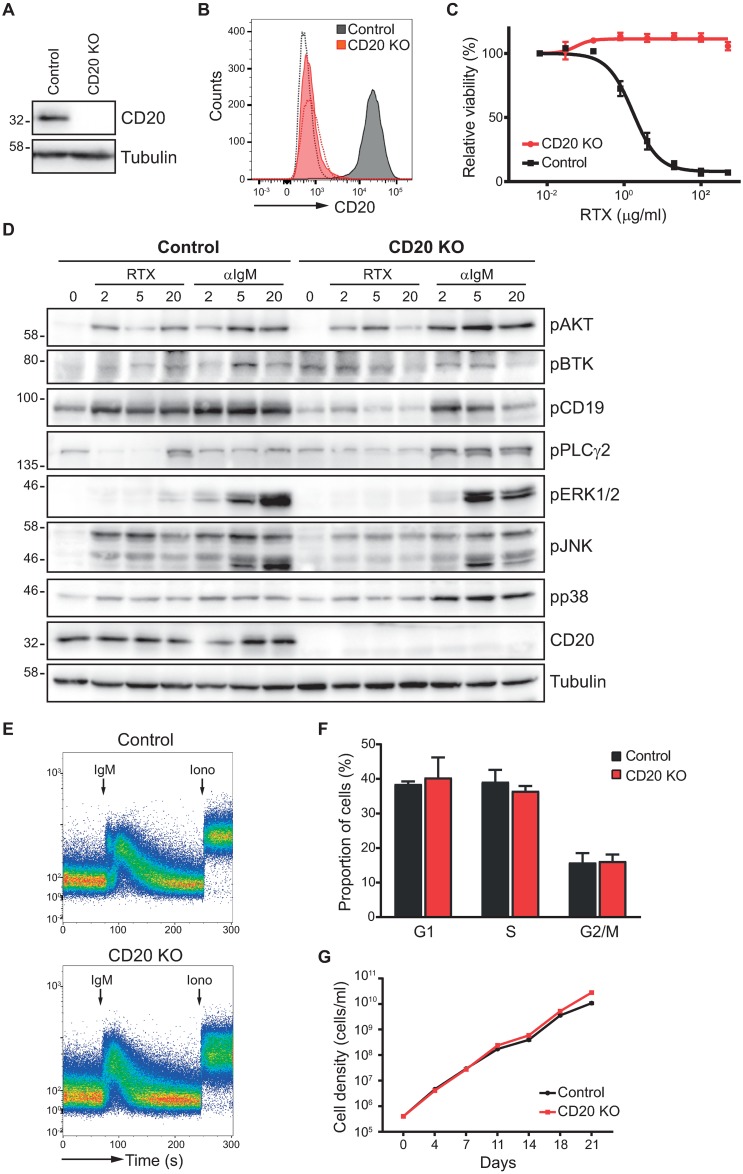
CD20 is dispensable for proper B-cell signaling and proliferation. (A) Lysates of Ramos control and CD20 knockout (CD20 KO) cells were immunoblotted with CD20 and tubulin antibodies. Cas9-expressing cells are used as control cells throughout the entire study. Molecular weight marker is shown on the left. (B) CD20 surface expression for Ramos control (black) and CD20 knockout (red) cells was determined by staining with CD20-APC antibody and detected by flow cytometry. Dashed lines represent staining of the respective cell line with isotype control antibody. (C) Dose-response curve of control (black) and CD20 knockout (red) Ramos cells against concentration range of CD20-antibody Rituximab (RTX). (D) Ramos control and CD20 knockout cells were stimulated with Rituximab (RTX) or anti-IgM antibodies for indicated times (minutes). A representative example (out of 4) of the western blotting with the indicated antibodies is shown. Molecular weight marker is shown on the left. Quantification of band intensities from replicate experiments is summarized in S3 Fig. (E) Ramos cells were loaded with the Fluo-4 calcium indicator and the influx of calcium in response to αIgM and ionomycin was determined by flow cytometry. Arrows indicate the time points of stimuli addition. (F) The proportion of Ramos control or CD20 knockout cells in the individual phases of the cell cycle were determined by staining with propidium iodide and measuring the DNA content by flow cytometry. Percentage of cells in G1, S or G2/M phase was evaluated by FlowJo. Average of three independent replicates plus SD is shown. (G) Cell growth curve for Ramos control and knockout cells was measured during the period of 21 days. N = 3 (mean ± SD (negligible, unseen behind the points)).

In order to thoroughly analyze the functional consequences of CD20 deficiency, we employed CRISPR/Cas9 technology to fully delete CD20. We targeted the *MS4A1* gene (encoding CD20) with a CRISPR guideRNA in two different human B-cell lines representing distinct B-cell malignancies, Ramos (Burkitt lymphoma) and MEC1 (CLL-like). Lentiviral constructs carrying cloned guideRNAs were transduced into the target cells and CD20-negative cells were then sorted out by fluorescence-activated cell sorting. We have fully abrogated CD20 expression at both mRNA and protein level in both cell lines (Fig 1A and 1B and S1 Fig). We ruled out undesired editing in any of the top predicted potential off-target genes through Sanger sequencing. To functionally evaluate the loss of CD20, we assessed the response of CD20-knockout cells to the therapeutic CD20 antibodies Rituximab (RTX) or Obinutuzumab (OBI). One of the mechanisms of action of CD20 mAbs beside direct cell lysis and antibody-dependent cell lysis is a complement-dependent cytotoxicity [21]. In this case, the mAbs bound on the target cells activate complement cascade, which consequently results in the lysis of these marked cells. We therefore mixed the cells with RTX or OBI mAbs in the presence of human serum as the source of complement system and assessed the killing efficiency. As expected, CD20-knockout cells were found to be completely resistant (Fig 1C and S2 Fig).

We then wanted to address CD20 involvement in the regulation of BCR signaling. BCR triggering initiates a well-defined and complex series of signaling events leading to phosphorylation of adaptor proteins like CD19, phosphorylation and thereby activation of various kinases like BTK, AKT, activation of phospholipase C, induction of calcium flux and activation of mitogen-activated protein (MAP) kinases [22]. This all ultimately results in the activation of specific transcription factors (e.g. NFAT, NFκB). We therefore investigated the effect of CD20 loss upon the activation of individual BCR signaling proteins in both Ramos and MEC1 cell lines in response to the triggering of IgM-BCR as the major type of B-cell receptor (Fig 1D and S3 and S4 Figs). Surprisingly, CD20 deficiency did not result in any dramatic defects in BCR signaling. There seemed to be a slight increase in the activation of proximal signaling proteins AKT and PLCγ2 in Ramos CD20-knockout cells, however, this was not maintained across multiple experiments and these changes were not significant (S3 Fig). Furthermore, they were not paralleled in the MEC1 cell line (S4 Fig). The activation of MAP kinases ERK1/2 and JNK was overall only partly decreased, while p38 activity seemed higher in Ramos cells, although again not very reproducibly (S3 Fig). The only consistently observed defect thus appeared only the deficient phosphorylation of CD19 in the Ramos knockout cells.

Taken together, CD20 knockout cells did not show any dramatic alterations to the BCR stimulation by anti-IgM that would be consistent across samples and experiments, but conversely responded relatively similarly as their unedited counterparts. Notably, RTX treatment alone also did activate the majority of these signaling proteins in unedited cells, suggesting that RTX exploits some of the major downstream BCR signaling proteins (Fig 1D). This phenomenon was not unique to RTX itself, but could be reproduced also when stimulating wildtype cells with other CD20 mAbs Ofatumumab and Obinutuzumab (S5 Fig).

As CD20 was proposed to primarily regulate calcium transport, we next analyzed the calcium flux in response to BCR stimulation in the absence of CD20 (Fig 1E). Surprisingly, the CD20-knockout Ramos cells were fully proficient and showed no defect in comparison to control cells. Since CD20 was suggested to be involved in the calcium influx across the plasma membrane as opposed to the intracellular stores, we pretreated the cells with the calcium chelator EGTA to assess BCR-triggered calcium release solely from the intracellular sources and subsequently added extracellular calcium to measure the integrity of extracellular influx separately. We did not observe any defect in neither intracellular nor extracellular flux of calcium in the absence of CD20 (S6 Fig). Hence, CD20 does not seem to function as any essential calcium channel regulator in the BCR-triggered response in malignant cells.

Next, we explored the proliferative capabilities of CD20 knockout Ramos and MEC1 cells. We did not observe any changes in the cell cycle progression (Fig 1F and S7A Fig) or cell division between the control and CD20 knockout cells (Fig 1G and S7B Fig). In summary, there were no alterations in any of the examined key processes occurring in either of the generated CD20-knockout B cell lines.

**Fig 2:**
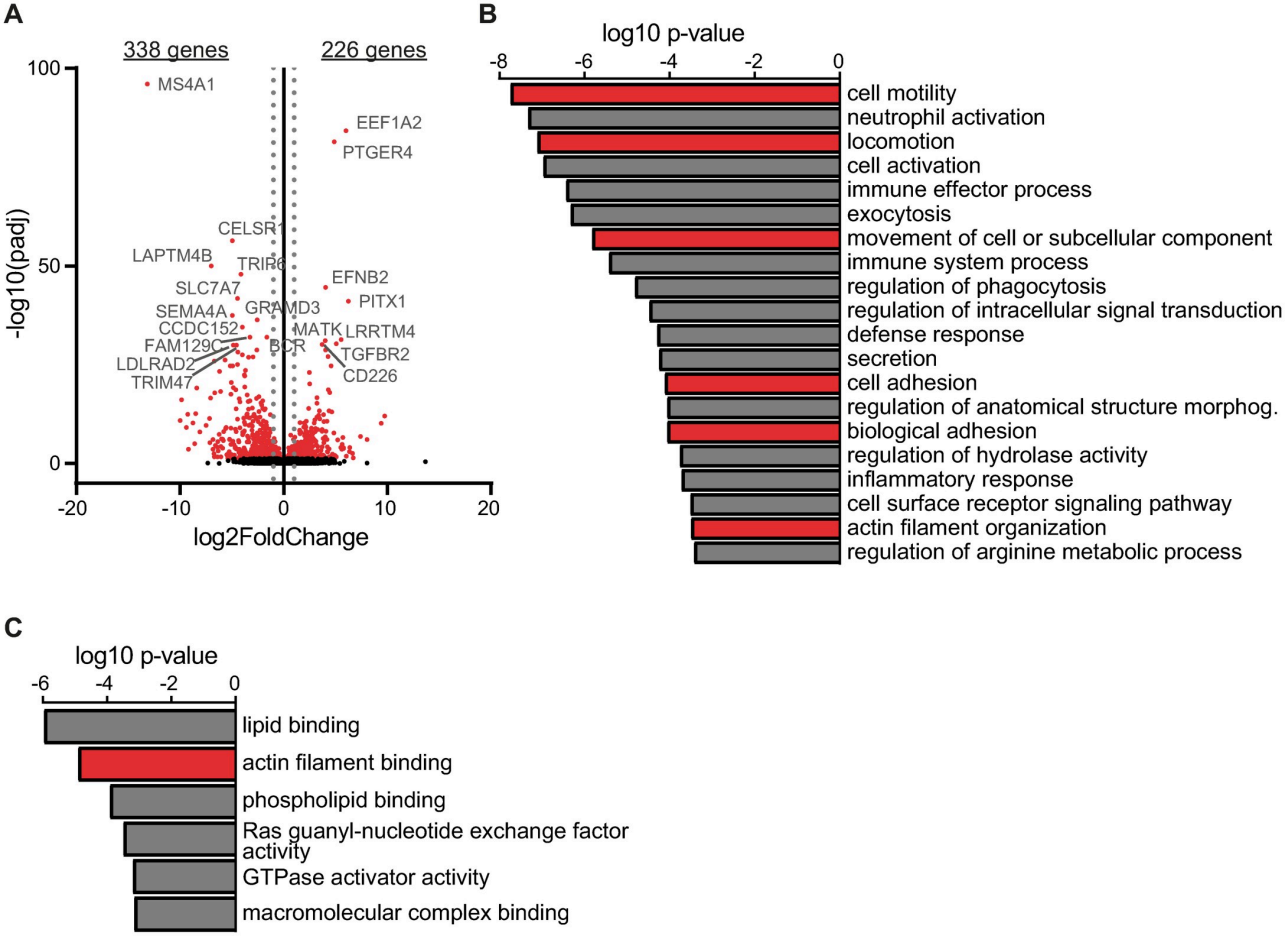
RNA sequencing reveals depletion of genes involved in motility and adhesion. (A) Volcano plot showing differentially expressed genes in MEC1 CD20 knockout cells relative to their control counterparts. Number of genes downregulated or upregulated in CD20 KO cells is shown on the top (fold change > 2; adjusted p-value < 0.05). Red dots mean significant genes (adjusted p-value < 0.05). Top 20 genes (by adjusted p-value) are indicated. (B) Gene ontology enrichment calculated with GOrilla online tool from results in A showing the top 20 depleted biological processes. (C) Gene ontology enrichment calculated from results in A showing significantly depleted molecular functions (log10 p-value < –3) as calculated by GOrilla online tool.

In order to unbiasedly portray the intracellular changes triggered by CD20 loss, we isolated RNA from both control and knockout cells and subjected it to RNA sequencing. Differential gene expression analysis of MEC1 cell lines revealed 338 genes significantly downregulated and 226 genes significantly upregulated in the CD20 knockout cells (Fig 2A). Interestingly, only 85 genes were found downregulated and just 7 genes upregulated in Ramos CD20 knockout cells (S8 Fig). *MS4A1* gene itself was naturally by far the most downregulated gene in both cell lines. To pinpoint the pathways that are mostly altered in the knockout cells, we performed a gene set enrichment analysis (Fig 2B and 2C and S9 Fig). Note that the limited number of differentially expressed genes in Ramos cells did not allow for any reasonable gene ontology analysis. On the other hand, general biological processes like cellular signaling, immune cell activation and immune effector processes were diminished in MEC1 knockout cells. To our surprise, however, we also identified multiple gene ontology (GO) terms related to actin filament, cell motility and cell adhesion among the top 20 most significantly downregulated biological processes (Fig 2B). In addition, actin filament binding scored high among the top depleted GO terms regarding functional annotations (Fig 2C).

**Fig 3:**
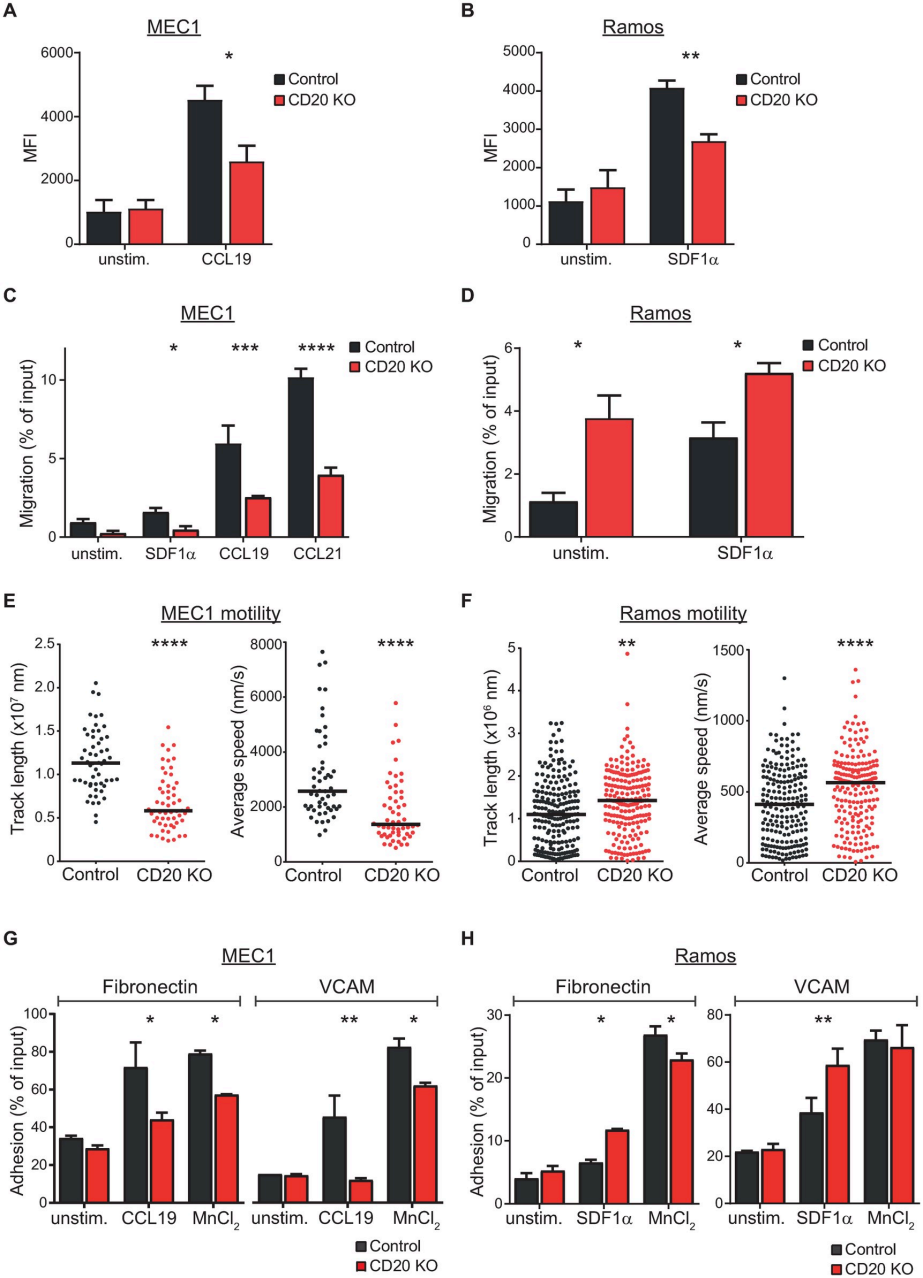
CD20 is required for proper actin polymerization, migration and adhesion. (A) MEC1 and (B) Ramos control and CD20 knockout cells were stimulated for 15s with CCL19 or SDF1α, respectively, fixed and stained with TRITC-phalloidin to visualize polymerized actin. Mean fluorescence intensity (MFI) measured by flow cytometry is shown as the mean + SD of three independent biological replicates. (C) Transwell migration of MEC1 control and CD20 knockout cells to the gradient of SDF1α, CCL19 and CCL21 chemokines. Percentage of migrated cells related to the total input cells is shown. N = 3 (mean+SD) (D) Transwell migration of Ramos control and CD20 knockout cells to the gradient of SDF1α chemokine. Percentage of migrated cells related to the total input cells is shown. N = 3 (mean+SD) (E) Cell motility of MEC1 control (black) and CD20 knockout (red) cells was monitored by time-lapse microscopy for 1 hour. Total track length and average speed were determined by CellTracker software for over 40 cells. Horizontal bars represent median value. Data for a representative experiment is shown (n = 2), p-value < 0.0001 (F) Cell motility of Ramos control (black) and CD20 knockout (red) cells was monitored by time-lapse microscopy for 1 hour. Total track length and average speed were determined by CellTracker software. Data are combined from four independent experiments. Horizontal bars represent median value, *** p<0.001, **** p<0.0001. (G) Cell adhesion of MEC1 cells onto the layer of fibronectin (left part) or VCAM (right part). Integrins were first activated by stimulating the cells with CCL19 or MnCl_2_ for 15min. Percentage of adhered cells versus total input cells is shown for a representative experiment (n = 3, mean+SEM), * p<0.05, ** p<0.01 (H) Cell adhesion of Ramos cells onto the layer of fibronectin (left part) or VCAM (right part). Integrins were first activated by stimulating the cells with SDF1α or MnCl_2_ for 15min. Percentage of adhered cells versus total input cells is shown for a representative experiment (n = 3, mean+SEM), ** p<0.01.

The observation that CD20 may play a role in actin cytoskeleton organization and actin-dependent biological processes like migration and adhesion was very intriguing and unprecedented. CD20 may thus be involved in chemokine-triggered cellular readouts instead of the proposed BCR-induced signaling. To investigate this further, we have selected three homeostatic chemokines (SDF1α (CXCL12), CCL19 and CCL21), which are responsible for navigating the cells throughout the body and their homing into lymphoid tissues under resting conditions [23]. We first stimulated the cells with the chemokine SDF1α or CCL19 and assessed the actin polymerization propensity. Note that MEC1 cells are basically lacking the SDF1α receptor CXCR4, while being positive for CCR7, the receptor for CCL19 and CCL21 chemokines. In contrast, Ramos cells are positive for CXCR4, but have undetectable expression of CCR7 (S10 Fig). Strikingly, both MEC1 and Ramos cells were clearly defective in proper actin filament polymerization in response to the respective chemokine, when CD20 was absent (Fig 3A and 3B). Since actin cytoskeleton reorganization is crucial for appropriate response to chemokines, we investigated the migratory capacities towards individual chemokines in a transwell migration experiment. MEC1 knockout cells displayed a strong impairment in their ability to migrate towards SDF1α, CCL19 or CCL21 (Fig 3C). Naturally, MEC1 cells responded only very poorly to SDF1α chemokine due to stated marginal expression of its receptor CXCR4 (S10 Fig). Interestingly, we observed strong and reproducible basal migration activity in Ramos knockout cells without any stimulus (Fig 3D). Although they were capable of only marginal further activation of migration, the migration of Ramos CD20 knockout cells in response to SDF1α chemokine was still more than 60% higher than the control counterparts.

To further investigate the effect of CD20 loss upon the cellular motility, we subjected the cells to a time-lapse live cell microscopy. Imaging the cells for over one hour revealed that MEC1 CD20 knockout cells are indeed less mobile, as shown by a strong reduction in their average speed as well as total length of the trajectory traveled (Fig 3E and S11A Fig). Ramos CD20-deficient cells, in contrast, exhibited enhanced spontaneous motility (Fig 3F and S11B Fig), explaining the enhanced basal transwell migration observed previously. In addition, we assessed the adhesion properties to two different matrices, fibronectin and VCAM1. We observed strong defects in chemokine-triggered adhesion as well as reduction in manganese ions-triggered (so called outside-in) integrin activation in MEC1 knockout cells (Fig 3G). Ramos cells, however, demonstrated increased adhesion in response to SDF1α when CD20 was missing (Fig 3H).

The discrepancy in migration and adhesion capabilities of Ramos versus MEC1 CD20 knockout cells was puzzling. It might to some extent be explained by the different nature of the cells, Ramos line representing Burkitt lymphoma, while MEC1 cells originating from chronic B-cell leukemia. Indeed, the gene expression profiles revealed by RNA sequencing demonstrate strong differences between these two cell lines with over 4.000 genes more than 2-fold differentially expressed (S12A and S12B Fig). Consequently, expression changes triggered by CD20 deletion show very poor correlation between Ramos and MEC1 cells (S12C Fig).

Interestingly, CD20 antibodies were shown to trigger homotypic adhesion of cells, a unique adhesive event induced by cell activation. Hence, CD20 was proposed to play a role in the regulation of homotypic adhesion in general [24]. We have thus investigated the effect of CD20 knockout on homotypic adhesion induced by other antibodies like anti-HLA-DR and anti-CD44, two well-known inducers of homotypic adhesion [24, 25]. However, we found no defects in this process in neither of the studied knockout cells (S13 Fig).

**Fig 4:**
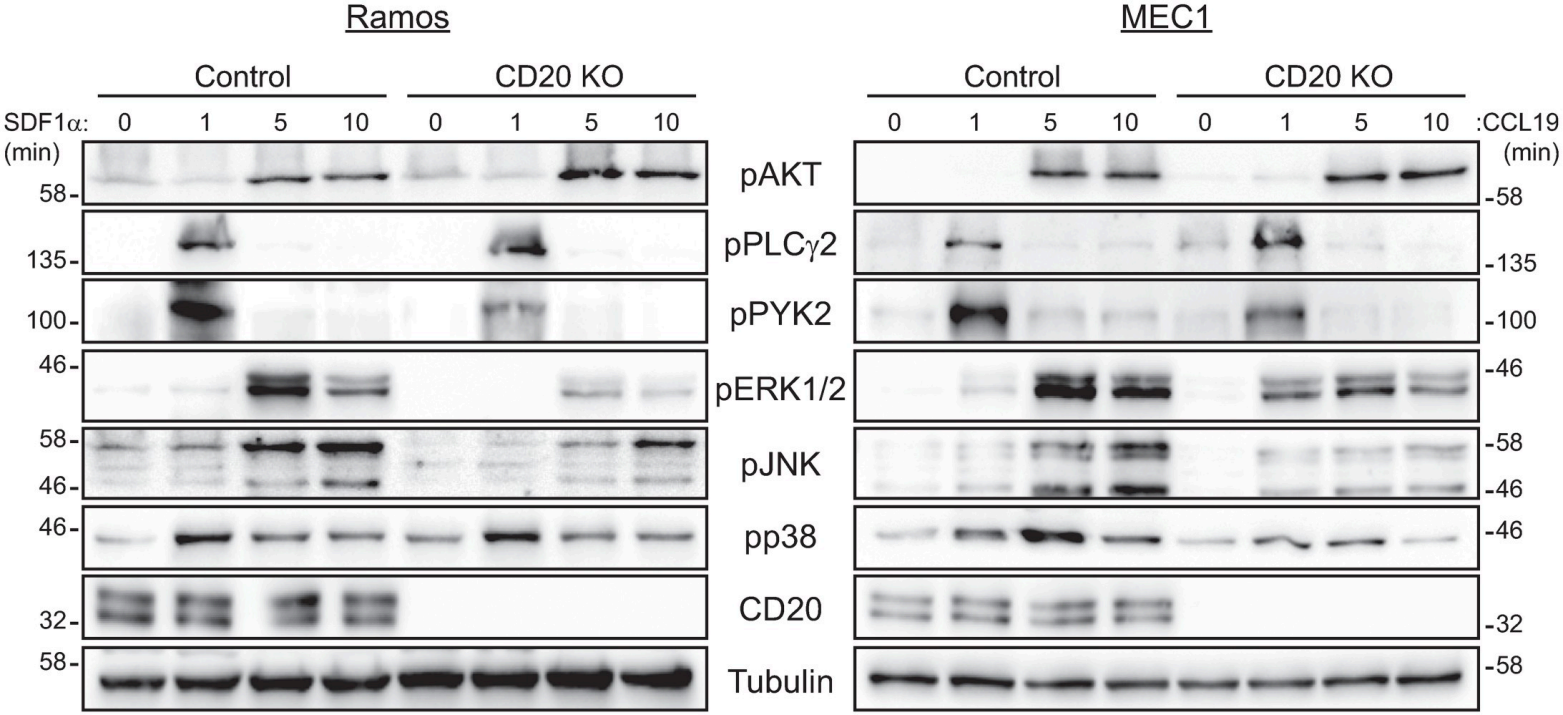
Defective chemokine signaling in the absence of CD20. Ramos (left panels) and MEC1 (right panels) control and CD20-knockout cells were stimulated with chemokines SDF1α or CCL19, respectively, for indicated times (minutes). A representative example (out of 3) of the western blotting with the indicated antibodies is shown. Molecular weight marker is shown on the left and right of the panels, respectively. Quantification of band intensities from replicate experiments is summarized in S14 Fig.

To understand the molecular mechanism behind altered chemokine responses, we have investigated the activation of individual signaling proteins in response to chemokine stimulation (Fig 4 and S14 Fig). The activation of proximal proteins AKT and PLCγ2 was equal in both control and CD20 knockout cells. However, we found a strong defect in the activation of PYK2 as well as strongly diminished activation of downstream MAP kinases ERK1/2 and JNK, in both Ramos and MEC1 cell lines.

**Fig 5:**
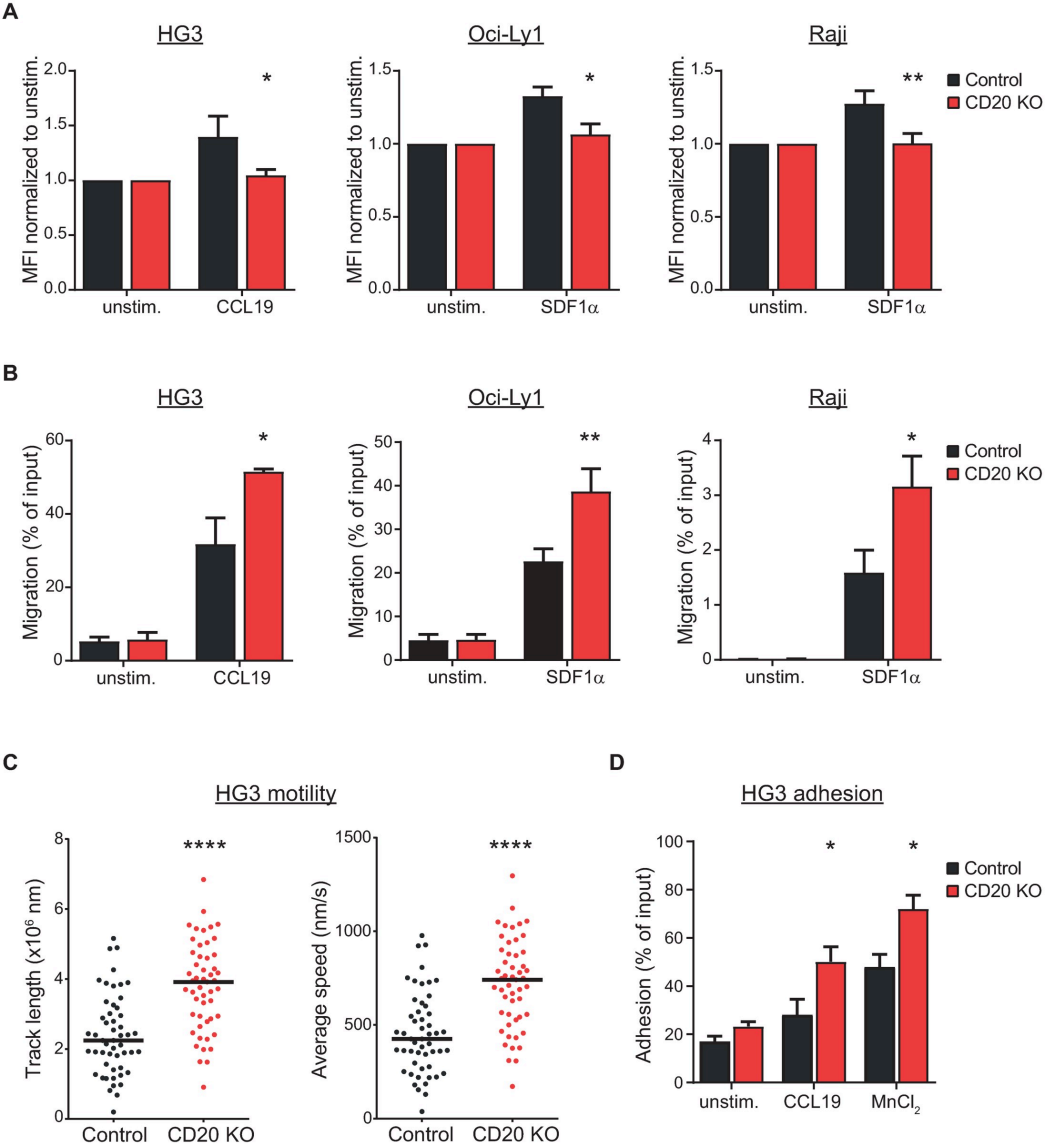
Defective actin polymerization, enhanced migration and adhesion in multiple CD20 knockout cell lines. (A) HG3, Oci-Ly1 and Raji control and CD20 knockout cells were stimulated for 15s with CCL19 or SDF1α, respectively, fixed and stained with TRITC-phalloidin to visualize polymerized actin. Mean fluorescence intensity (MFI) measured by flow cytometry for the stimulated samples was normalized to the unstimulated counterparts and is shown as the mean + SD of three independent biological replicates. (B) Transwell migration of HG3, Oci-Ly1 and Raji control and CD20 knockout cells to the gradient of CCL19 or SDF1α chemokines, respectively. Percentage of migrated cells related to the total input cells is shown as an average from 3 independent replicates (mean+SD) (C) Cell motility of HG3 control (black) and CD20 knockout (red) cells was monitored by time-lapse microscopy for 1 hour. Total track length and average speed were determined by CellTracker software for over 40 cells. Horizontal bars represent median value. Data for a representative experiment is shown (n = 3), p-value < 0.0001 (D) Cell adhesion of HG3 cells onto the layer of fibronectin. Integrins were first activated by stimulating the cells with CCL19 or MnCl_2_ for 15min. Percentage of adhered cells versus total input cells is shown for a representative experiment (n = 3, mean+SEM), * p<0.05.

To shed more light on the role of CD20 in the actin regulation, migration and adhesion, we have expanded the portfolio of our CD20 knockout cell lines by generating additional CRISPR-mediated CD20 deletions in HG3 cell line (CLL), Oci-Ly1 cell line (diffuse large B-cell lymphoma) and Raji cell line (Burkitt lymphoma) (S15 Fig). We could demonstrate that CD20 loss results in defective actin polymerization also in all three newly generated cell lines (Fig 5A). Transwell migration assays revealed strongly enhanced migration in the absence of CD20 (Fig 5B). We have then subjected the HG3 cell line to the time-lapse live cell imaging to conclude that the CD20 knockout cells are indeed more mobile (Fig 5C and S11C Fig). Finally, adhesion assay revealed that HG3 CD20 knockout cells are also more adhesive to fibronectin matrix (Fig 5D). Taken together, we could show with five different malignant B-cell lines that CD20 loss has profound consequences upon the actin polymerization, proper mobility of the cells and their proper adhesion. While adhesion and migration were enhanced in four of the cell lines, they were reduced in MEC1 cells. It is currently unclear why MEC1 cells behave so differently from the other cell lines. This is an interesting observation, which would deserve further extensive investigation as to the nature of the intracellular differences among the cell lines associated with the signaling pathways regulating adhesion and migration processes.

**Fig 6:**
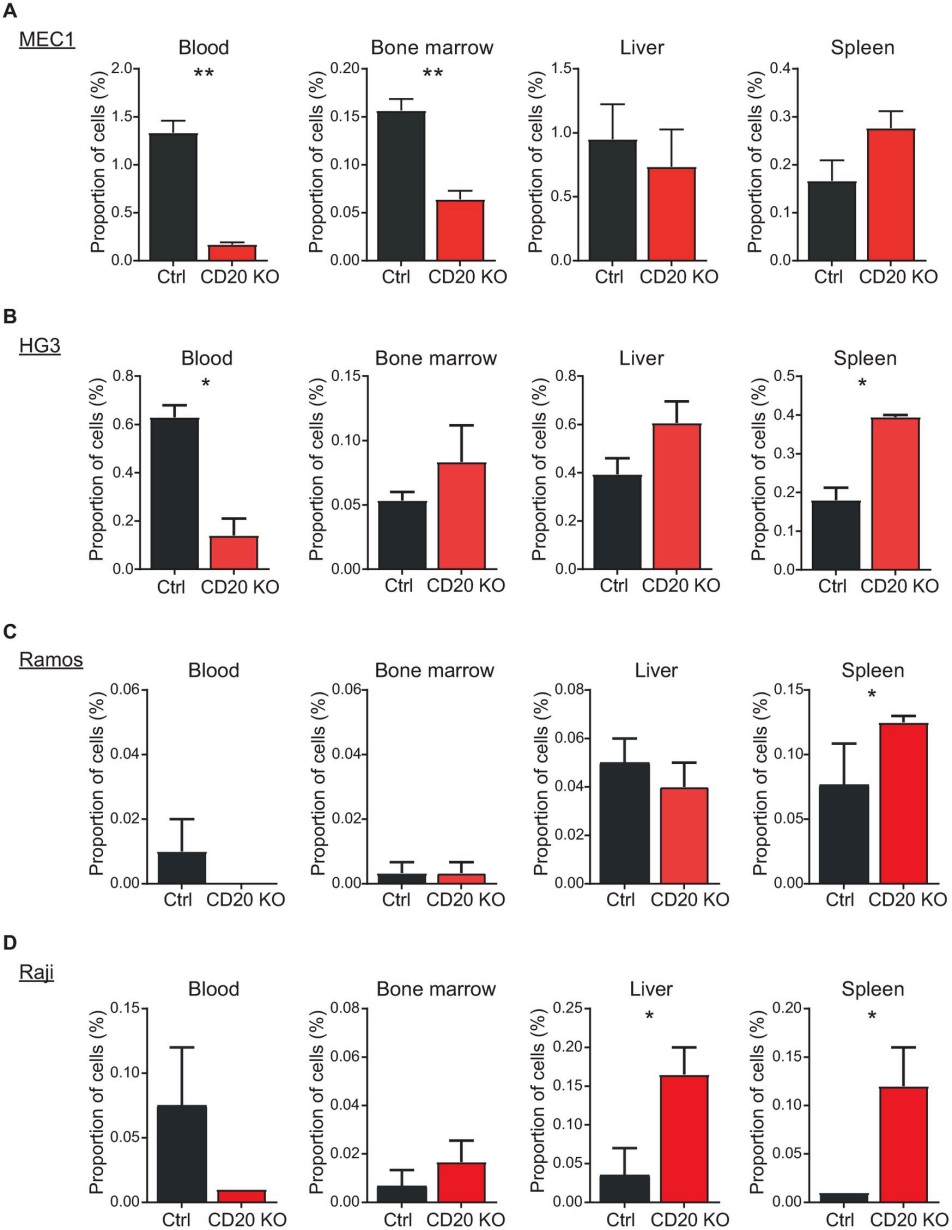
Defective *in vivo* cell homing in the absence of CD20. (A) Calcein-loaded MEC1 control and CD20-knockout cells were intraperitoneally injected into NSG mice (3 mice each) and the proportion of calcein-positive cells in spleen, liver, blood and bone marrow 24 hours later was determined by FACS. A representative experiment is shown (mean+SEM calculated from individual mice together; n = 3). ** p<0.01. (B) Calcein-loaded HG3 control and CD20-knockout cells were injected into the tail vein of the NSG mice (3 mice each) and the proportion of calcein-positive cells in spleen, liver, blood and bone marrow 6 hours later was determined by FACS. A representative experiment is shown (mean+SEM calculated from individual mice together; n = 3). * p<0.05. (C) Ramos control and CD20-knockout cells were injected intraperitoneally and processed as in A. (D) Raji control and CD20-knockout cells were intraperitoneally injected into NSG mice (3 mice each) and processed as in A.

Finally, the altered response to chemokines suggests that there might be a defect in proper homing of CD20-deficient cells *in vivo* into the lymphoid compartments. We have thus injected MEC1 control and CD20-knockout cells intraperitoneally into NOD-Scid-IL2Rγnull (NSG) mice and assessed 24 hours later their presence in blood, spleen, liver and bone marrow (Fig 6A). While we found increased retaining of CD20-knockout cells in the spleen (although not significant), there was a dramatic drop in their recruitment into the blood and bone marrow. We have then repeated the *in vivo* experiment using our CD20 knockout variants of HG3 (Fig 6B), Ramos (Fig 6C) and Raji cell lines (Fig 6D), which were injected either intraperitoneally (Ramos, Raji) or intravenously into the tail vein (HG3). All these cell lines demonstrated strong accumulation of CD20 knockout cells within the spleen, while strongly reduced presence in blood. These data confirm disturbed recirculation of CD20-deficient cells *in vivo* in comparison to the control cells.

In summary, our observation that CD20 is entirely dispensable for BCR signaling and B-cell proliferation in malignant B cells is fully in line with the findings from the CD20-knockout mice, where no defects in the activation of individual BCR signaling proteins and normal cell proliferation were described [12, 13]. Further endorsement comes from a report of a patient with homozygous mutation in *CD20* gene, resulting in complete lack of CD20 protein expression, where normal BCR activation, intact calcium flux and unaffected B cell proliferation was detected [26]. Instead, we uncovered the role of CD20 in actin-dependent cellular processes. In this regard, actin cytoskeleton regulation was recently found to be associated with CD20 through an approach based on similar gene expression profiles [27]. Interestingly, CD20 signals were also shown to be triggered via heterotrimeric G-proteins [28]. Since CXCR4 and CCR7 are G-protein coupled receptors, it is plausible that their triggering may induce activation of CD20. In fact, SDF1α was shown to enhance CD20 expression [29], suggesting that high CD20 levels are required in a feed-forward loop to properly maintain the response to the chemokine. Once CD20 is deleted, there is a defect in actin polymerization and a misregulation of induced migration and adhesion. The direction of this misregulation is possibly inherent to a particular cell line and is most likely affected by many other specific factors modulating the overall response.

We could identify PYK2 as a possible candidate protein mediating the effects of CD20 signaling. PYK2 belongs to the focal adhesion family of nonreceptor protein tyrosine kinases [30]. Interestingly, PYK2 was shown to be activated in response to intracellular increase of calcium levels triggered by activation of surface receptors like G-protein coupled receptors or integrins [31]. Note that CD20 was in fact proposed to function as a calcium channel [6, 7]. Activated PYK2 then regulates MAP kinase signaling pathways as well as actin cytoskeleton remodeling, adhesion and migration [32, 33]. Importantly, PYK2-deficient murine macrophages show defect in migration towards a chemokine gradient, defective cytoskeleton reorganization in response to chemokines and altered cell adhesion [34]. Hence, the same functions are affected as observed in our CD20 knockout cells.

In conclusion, our data suggest that CD20 is directly involved in chemokine-triggered adhesion and migration and it may thereby regulate the recirculation of peripheral blood B cells into the spleen, bone marrow and lymph nodes and their interaction with the stromal cells.

## Supporting information

S1 Fig Validation of CD20 deletion by CRISPR/Cas9. (A) Quantitative real-time PCR was performed for Ramos and MEC1 cells infected with either mock or CD20 sgRNA construct. Relative CD20 mRNA expression in knockout (CD20 KO) versus control (Ctrl) cells is shown (mean+SD, n = 3). (B) Western blotting of MEC1 control and knockout cells demonstrates complete loss of CD20 protein in the knockout line. Molecular weight marker (in kDa) is shown on the left. (C) CD20 surface expression on MEC1 control (black) and CD20 knockout (red) cells was determined by staining with CD20-APC antibody and detected by flow cytometry. Dashed lines represent isotype control antibody staining of the respective cell line.
(EPS)

S2 Fig Ramos CD20 knockout cells are fully resistant to CD20 monoclonal antibody Obinutuzumab (OBI). Dose-response curve of control (black) and CD20 knockout (red) Ramos cells against concentration range of CD20-antibody Obinutuzumab (OBI) is shown (mean±SD, n = 3).
(EPS)

S3 Fig Quantification of Western blots from Fig 1D. Band intensities for individual phospho-specific antibodies were determined by ImageJ and normalized against tubulin. Average ± SEM from three independent replicates is shown.
(EPS)

S4 Fig CD20 is dispensable for proper B-cell receptor signaling in MEC1 cell line. (A) MEC1 control and CD20 knockout cells were stimulated with anti-IgM antibody for indicated times (minutes). A representative example (out of 4) of the western blotting with the indicated antibodies is shown. Molecular weight marker (in kDa) is shown on the left. (B) Band intensities for individual phospho-specific antibodies in A were determined by ImageJ and normalized against tubulin. Average ± SEM from three independent replicates is shown.
(EPS)

S5 Fig CD20 mAbs trigger unique activation of BCR signaling proteins. Wildtype Ramos cells were stimulated with anti-IgM antibody or anti-CD20 antibodies Rituximab (RTX), Ofatumumab (OFA) or Obinutuzumab (OBI) for indicated times (minutes). A representative example of the western blotting with the indicated antibodies is shown. Molecular weight marker (in kDa) is shown on the left. Phospho-SFK represents activatory residue (Y416) of Src-family kinases (SFK), whereas phospho-LYN marks the inhibitory residue (Y507) within LYN and/or other SFKs.
(EPS)

S6 Fig CD20-deficient cells display normal calcium flux from intracellular stores and normal influx across the plasma membrane. Ramos cells were loaded with the Fluo-4 calcium indicator and pretreated with EGTA. The release of calcium from intracellular stores was triggered by the addition of αIgM antibody. Addition of extra calcium ions into the media assessed the calcium influx across the plasma membrane. Flow cytometry measurement for a representative experiment is shown. Arrows indicate the time points of stimuli addition.
(EPS)

S7 Fig MEC1 CD20 knockout cells display normal progression through the cell cycle and normal cell growth. (A) Proportion of MEC1 knockout or control cells in individual phases of the cell cycle was determined by staining with propidium iodide and measuring the DNA content by flow cytometry. Percentage of cells in G1, S or G2/M phase was evaluated by FlowJo. Average of three independent replicates plus SD is shown. (B) Cell growth curve for MEC1 knockout and control cells was measured during the period of 14 days (mean ± SD (negligible, unseen behind the points); n = 3).
(EPS)

S8 Fig Differential gene expression analysis in Ramos cells. Volcano plot showing differentially expressed genes in Ramos CD20 knockout cells relative to their control counterparts. Number of genes downregulated or upregulated in CD20 KO cells is shown on the top (fold change > 2; adjusted p-value < 0.05). Red dots mean significant genes (adjusted p-value < 0.05). Top 20 genes (by adjusted p-value) are indicated.
(EPS)

S9 Fig Gene set enrichment analysis in MEC1 showing upregulated gene ontology terms. (A) Gene ontology enrichment calculated from RNA sequencing results in Fig 2A showing the top 20 upregulated biological processes. (B) Gene ontology enrichment calculated from RNAseq results in Fig 2A showing significantly upregulated molecular functions (log10 p-value < –3).
(EPS)

S10 Fig Surface expression of chemokine receptors CXCR4 and CCR7 on control and CD20 knockout cells. MEC1 (A) and Ramos (B) control and CD20 knockout cells were stained with antibodies against CXCR4 (top panels) and CCR7 (bottom panels) and were assessed by flow cytometry. Dashed lines represent samples stained with isotype control antibody (neg.), filled histograms represent cells stained with specific antibodies.
(EPS)

S11 Fig Time-lapse microscopy of MEC1, Ramos and HG3 cells. (A) Cell motility of MEC1 control (black) and CD20 knockout (red) cells was monitored by time-lapse microscopy for 1 hour. Average distance from the origin was determined by a CellTracker software for over 40 cells. Horizontal bars represent median value. Data for a representative experiment is shown (n = 2), ns = not significant. (B) Cell motility of Ramos control (black) and CD20 knockout (red) cells was monitored by time-lapse microscopy for 1 hour. Average distance from the origin is shown as determined by CellTracker software. Data are combined from four independent experiments. Horizontal bars represent median value, ** p<0.01. (C) The same as in A is shown for HG3 control and CD20 knockout cells (n = 3).
(EPS)

S12 Fig Correlation of gene expression profiles between MEC1 and Ramos cell lines. Gene expression of control (A) or CD20-knockout (B) cell lines was determined by RNA sequencing. Normalized counts are plotted for Ramos versus MEC1 cells and correlation coefficient is shown. Numbers depict number of genes upregulated more than 2-fold in MEC1 or Ramos cell lines, respectively. (C) Log2 fold change of gene expression altered in CD20-knockout over control cells is correlated for Ramos versus MEC1 cell lines. MS4A1 gene is marked as the most depleted gene common to both cell lines.
(EPS)

S13 Fig Homotypic adhesion is not altered in CD20-knockout cells. Control and CD20-knockout cells were left untreated or incubated with CD44 or HLA-DR antibodies for 6h (MEC1) or 24h (Ramos). Representative examples of photographs of induced cell clustering are shown for both MEC1 (A) and Ramos (B) cells.
(EPS)

S14 Fig Western blot quantification for Fig 4. Band intensities for individual phospho-specific antibodies were determined by ImageJ and normalized against tubulin. Average ± SEM from three independent replicates is shown for Ramos (A) and MEC1 (B) cell line.
(EPS)

S15 Fig Surface expression of CD20, chemokine receptors CXCR4 and CCR7 on control and CD20 knockout cells. (A) CD20 surface expression on HG3, Oci-Ly1 and Raji control (black) and CD20 knockout (red) cells was determined by staining with CD20-APC antibody and detected by flow cytometry. Dashed lines represent isotype control antibody staining of the respective cell line. HG3 (B), Oci-Ly1 (C) and Raji (D) control and CD20 knockout cells were stained with antibodies against CXCR4 (left panels) and CCR7 (right panels) and were assessed by flow cytometry. Dashed lines represent samples stained with isotype control antibody (neg.), filled histograms represent cells stained with specific antibodies.
(EPS)
